# Older Adults’ Communication Channel Profiles: Correlates & Implications for Protective Effects of Social Support

**DOI:** 10.1093/geroni/igaf122.1348

**Published:** 2025-12-31

**Authors:** JungYeon Suh, Ha Young Choi, Shannon Mejia

**Affiliations:** University of Illinois Urbana-Champaign, Champaign, Illinois, United States; University of Illinois Urbana-Champaign, Urbana, Illinois, United States; University of Illinois Urbana-Champaign, Champaign, Illinois, United States

## Abstract

Social support is essential to health and well-being in later life. Age-related declines in social network size heightens the importance of social support. In this study we examine the implications of Information and Communication Technology (ICT) for the protective effects of social support. Using the 2016/2018 Health and Retirement Study (n = 11,068; aged 51-102), we employed latent profile analysis to identify four communication profiles: (1) email dominant (23.4%); (2) SNS dominant (13.4%); (3) in-person/phone contact (41.1%); and (4) high interaction across all channels (22.3%). Multinomial logistic regression and path analyses were conducted to identify sociodemographic predictors (age, gender, education, living alone status, and emotional and geographical closeness) of profile membership and how the profiles moderate relationships between social support and well-being (life satisfaction and loneliness). Compared to the email dominant profile, SNS profile tended to be women, younger, more educated, have more emotionally and geographically close family, and fewer friends. Meanwhile, in-person/phone contact profile was more likely to be older, men, living alone, and have lower education, more emotionally close children, and family and friends who were geographically, but not emotionally close. The protective effect of support from children on well-being was strongest for the SNS dominant profile. Conversely, the email dominant profile dampened the effects of support from family and friends. Our findings imply that older adults adopt different communication strategies, shaping the protective effects of social support. Additionally, SNS interactions with specific social networks should be differentiated from general use, when assessing its effect on older adults’ well-being.

